# Commencements

**Published:** 1894-09

**Authors:** 


### COMMENCEMENTS.

### Boston Dental College.

The twenty-seventh annual commencement exercises of the Boston Dental College were held in Berkeley Temple, Boston, Mass., on Wednesday, June 20th, 1894, at 7:30 P. M.

The annual address was delivered by Rev. E. Winchester Donald, D.D.

The number of matriculates for the session was one hundred and fifty-two.

The degree of D.D.S. was conferred on the following graduates by I. J. Wetherbee, D.D.S., President of the College:

| NAME. | RESIDENCE. |
|---|---|
| B. DeF. Barton | New Brunswick |
| Harry G. Beekman | Massachusetts |
| Frank R. Chapman | Massachusetts |
| Harry P. Chase | New Hampshire |
| Peter A. Caulfield | Massachusetts |
| Charles H. Davis | Rhode Island |
| Carroll A. Egan | Massachusetts |
| Henry M. Egan | Massachusetts |
| Walter E. Farris | Massachusetts |
| Homere G. Fauteux | Prov. of Quebec |
| James J. Ford | Massachusetts |
| Harry C. Gleason | Maine |
| John E. Glidden | New Hampshire |
| Arthur W. Gould | Massachusetts |
| William T. Hill | Massachusetts |
| William R. Howard | Massachusetts |
| John H. Jackson | Washington |
| Harry P. Jones | Massachusetts |
| Walter E. Knapp | Massachusetts |
| George L. Mason | New Hampshire |
| Chas. A. McGinley | Massachusetts |
| Daniel A. Nason | Massachusetts |
| Wm. F. B. Reilley | New Hampshire |
| Wm. L. Rombough | Massachusetts |
| Louis W. Scott | Prov. of Quebec |
| Wyman H. Streeter | Massachusetts |
| Lucius K. Thayer | Massachusetts |
| Jos. L. F. Taylor | Maine |
| John B. Tufts | Massachusetts |
| Albert H. Ward | Massachusetts |
| Fred. G. Weeks | Maine |
| William J. Welch | Massachusetts |
| Cyrus R. Wetherell | Massachusetts |

Total, 33.

### East Tennessee Dental Association.

The annual meeting of the East Tennessee Dental Association will be held at Athens, Tenn., 13th, 14th and 15th of September, 1894.

A cordial invitation is extended to all members of the Dental Profession.

U. D. BILLMEYER, Chattanooga, President.

A. W. PALMER, Chattanooga, Secretary and Treasurer.

